# A proposed prognostic 7-day survival formula for patients with terminal cancer

**DOI:** 10.1186/1471-2458-9-365

**Published:** 2009-09-29

**Authors:** Jui-Kun Chiang, Ning-Sheng Lai, Mei-Huang Wang, Shi-Chi Chen, Yee-Hsin Kao

**Affiliations:** 1 Department of Family Medicine, Buddhist Dalin Tzu Chi General Hospital, Chiayi, Taiwan, Republic of China; 2 Department of Natural Biotechnology, Nanhua University, Chiayi County, Taiwan, Republic of China; 3 Department of Allergy, Immunology and Rheumatology, Buddhist Dalin Tzu Chi General Hospital, Chiayi, Taiwan, Republic of China; 4 Professor, School of Medicine, Tzu Chi University, Hualien, Taiwan, Republic of China; 5 Department of Nursing, Buddhist Dalin Tzu Chi General Hospital, Chiayi, Taiwan, Republic of China; 6 Department of Family Medicine, Tainan Municipal Hospital, Tainan, Taiwan, Republic of China; 7 Institute of Gerontology, College of Medicine, National Cheng Kung University. Tainan City, Taiwan, Republic of China

## Abstract

**Background:**

The ability to identify patients for hospice care results in better end-of-life care. To develop a validated prognostic scale for 7-day survival prediction, a prospective observational cohort study was made of patients with terminal cancer.

**Methods:**

Patient data gathered within 24 hours of hospital admission included demographics, clinical signs and symptoms and their severity, laboratory test results, and subsequent survival data. Of 727 patients enrolled, data from 374 (training group) was used to develop a prognostic tool, with the other 353 serving as the validation group.

**Results:**

Five predictors identified by multivariate logistic regression analysis included patient's cognitive status, edema, ECOG performance status, BUN and respiratory rate. A formula of the predictor model based on those five predictors was constructed. When probability was >0.2, death within 7 days was predicted in the training group and validation group, with sensitivity of 80.9% and 71.0%, specificity of 65.9% and 57.7%, positive predictive value of 42.6% and 26.8%, and negative predictive value (NPV) of 91.7% and 90.1%, respectively.

**Conclusion:**

This predictor model showed a relatively high sensitivity and NPV for predicting 7-day survival among terminal cancer patients, and could increase patient satisfaction by improving end-of-life care.

## Background

In hospice palliative care, an important issue is the ability to accurately predict the probability of survival of terminal cancer patients with short-term survival. Patients, family members, and providers may desire this information for their plans and decisions about "pain and distress;" clinicians may want to improve their prognostic skills [1,2]. When death is near, goals and plans of management often shift and a clinical pathway has been developed to guide care [3]. For example, patients expected to live for 1-2 weeks are incapable of oral fluid intake due to progressive cachexia and show a performance status of more than 3 points in the Eastern Cooperative Oncology Group (ECOG) scale. To improve general quality of life in these patients, simple hydration at 1000-1500 mL/day (400-600 kcal/day) is not recommended [4]. Prediction of short-term survival is important in the palliative care unit to improve quality of care at the end of life, in Taiwan as elsewhere.

Previous studies have shown that the accuracy of various prognostic scales is overoptimistic. A report of the meta-analysis suggested that survival of patients is typically 30% shorter than predicted in terminally ill cancer patients [5]. Another result from a systematic electronic literature search suggested that Clinical Prediction of Survival (CPS) is more than twice as likely to be overoptimistic versus overpessimistic, and to overestimate the length of actual survival by a factor of between 3 and 5 in advanced cancer patients [6]. Those scales are generated according to clinical symptoms and signs, which can vary considerably, although weakness and pain seem to be the most prevalent symptoms [7]. In several major studies, some laboratory parameters have been shown to be capable of providing additional prognostic information [8]. Nevertheless, a simple and accurate predictor for cancer patients is yet to be developed.

Despite a consensus for the need for survival prediction in patients with terminal cancer; both predictors selected and model vary widely. For established prognostic scales, accuracy varies from 0.61 to 0.82, decreasing with shorter survival prediction. Reuben's predicted survival table assesses terminal patients by six symptoms and signs: Karnofsky Performance Status (KPS), dyspnea, anorexia, dysphagia, xerostomia, and weight loss, with KPS the most important factor. Clinicians can predict a patient with the worst KPS score of surviving 16 days by 50% as well as 72 days by 10% if the other five factors exist. Accuracy for this model was not calculated [9]. Bruera's Poor Prognostic Indicator depends on three symptoms and signs as prognostic factors: dysphagia, cognitive barriers, and weight loss (refers to 10 kg weight loss within 6 months). Accuracy was calculated based on the PPV and NPV. When terminal patients meet these three prognostic factors, the anticipated survival time, within 4 weeks, is 74% accurate. The sensitivity of the model was 76%, specificity was 71%, PPV was 76%, NPV was 71%, [10]. Morita's Palliative Prognostic Index proposes five independent prognostic factors: physical function status (modified KPS), eating by mouth, edema, dyspnea, and delirium. When scores are more than six, survival time is less than three weeks with accuracy of 0.82. When scores are more than four, survival time is less than six weeks with accuracy of 0.77. Accuracy was calculated based on PPV and NPV [11]. Maltoni's Palliative Prognostic Score was developed from patient symptoms and signs, and complete blood count. This scale consists of six prognostic factors (e.g., dyspnea, anorexia, KPS, total white blood cell count and lymphocyte percentage, and clinical prediction of survival by experienced physicians). Maltoni found that the scale was as accurate as prediction by an experienced physician [12], with greatest accuracy from a combination of subjective prognostic judgements and objective validated tools [13].

Most recently, Chow's predictive model [14], Stone's prognostic scale [15], Chuang's prognostic score [16] and Bozcuk's intrahospital cancer mortality risk model [17] were developed to predict survival, and the latest three are for short-term survival. Of these, Chow's predictive model of the three-variable number of risk factors (NRF) -- nonbreast cancer, metastases other than bone and Karnofsky performance score <6_0 -- is clinically simple and user-friendly. For 3-month survival, this model estimates probability of patients with NFR ≤ 1, NFR = 2, NFR = 3 were 83%, 76%, and 44% respectively, in external validation set. In this model, all subjects were attending a palliative radiotherapy clinic. Stone's prognostic scale uses four variables: primary lung cancer, secondary liver cancer, C-reactive protein, and poor performance status (ECOG = 4) to predict 14-day survival. Stone's prognostic scale would have more value for identifying those patients not suitable for hospice admission, due to high predictive value (85%), and low positive predictive value (PPV) (39%). By contrast, Kelly's model suggested C-reactive protein as the simple prognostic indicator in patients with advanced cancer [18].

Chuang et al. constructed a prognostic scale with units including liver and lung metastasis, ECOG, weight loss, edema, cognitive impairment, fatigue, and ascites. Accuracy was directly derived from PPV and NPV. When the cutoff score was >6.0, PPV and NPV for patients with survival time < 1 week were 0.75 and 0.70 in the training set (accuracy, 0.72). For the testing set, values were 0.43 and 0.76, respectively (accuracy, 0.66) [10]. Bozcuk's intrahospital cancer mortality risk model predicted the probability of terminal cancer patients dying within 8 days. The scale was constructed based on predictors including ECOG score, duration of disease, emergency admission, Hb count and LDH level. Accuracy was expressed by ROC area (AUC). In the retrospective cohort, this model had an ROC area of 0.88 (*P *< 0.001, 95% CI: 0.84-0.92), whereas in the prospective cohort, it yielded an ROC area of 0.82 (*P *< 0.001, 95% CI: 0.73-0.90) [17].

Research has indicated that the rate of survival of less than 7 days in patients in hospice wards is about 15% [19]. In Taiwan, where patients with terminal cancer are referred to hospice for palliative care late in the course of their disease, the figure is approximately 30% [20,21]. In one Taiwanese study, only 3.5% of patients referred late in the course of the disease to hospice care lived longer than 180 days [21]; in Italy, a prospective cohort study showed that referral of cancer patients to palliative, end-of-life care tends to occur late in the course of the disease [22].

In Taiwan, between 2000 and 2004, the rates of late referrals for hospice care as a whole were 25.3%-28.9%, which were lower than the national statistics from the United State (35.1%), but higher than other published study reports (15.6%-20%) [23]. Furthermore, evidence suggests that late hospice referral increase the risk of a major depressive disorder in the first year of bereavement [24]. When patients were enrolled in hospice for less than 7 days, the hospice team often did not have adequate time to become familiar with patients and their home situation. The goal for comprehensive care (including the patients' wish such as to die at home) might become impossible to fulfill [25]. Part of the explanation for late referral can be attributed to difficulties in establishing an accurate prognosis [26].

In Taiwan, hospice care includes inpatient hospice care and hospice home care. In Taiwan, in 2004, overall utilization of hospice care is 15.4%, and hospice home care only is 2.6%, inpatient hospice care only is 10.1%, and both home care and inpatient care is 2.8%. Our study was inpatient hospice care [23].

Palliative care seeks to fulfill the needs of patients at the end of life. Ascertaining and carrying them out (e.g., the desire to die at home) requires the ability to predict short term survival. This study aimed to develop a validated model for 7-day survival prediction for terminal cancer patients to make more accurate prediction of short-term survival in order to facilitate timely referrals to palliative care as well as comprehensive care for terminally cancer patients.

## Methods

### Participants

We conducted a prospective observational cohort study for patients with terminal cancer. Of 727 terminal cancer patients from a hospice ward admitted from November 2004 to June 2007 in the Buddhist Dalin Tzu Chi General Hospital, the first 374 diagnosed with terminal cancer were chosen as the training group by which we would develop a prognostic measurement. The same measures were tested on the next 353 patients, the validation group. For the training group, number of admission during the study period was 415 cases, of which 374 cases were enrolled (91%). For the Validation group, the number of admissions during the study period was 430 cases, of which 353 cases were enrolled (82%). Overall percentage of enrollment was 86%. Most cases not enrolled were due to admission during a holiday or weekend.

Inclusion criteria were terminally ill cancer patients admitted to the palliative care unit at our hospital who were referred from other wards of the same hospital, from other hospitals or from home. All patients enrolled met with normative standards of hospice palliative enactment in Taiwan. Excluded were patients referred to other hospitals, since we could not accurately assess their complete records. Patient recruitment and study design were approved by the Institutional Review Board of Buddhist Dalin Tzu Chi General Hospital (Nos. B09303011 and B09502017). All patients gave their informed consent before being entered into the study. For those unconscious patients, we obtained proxy consent from relatives.

### Data collection

Data on demographics, clinical symptoms and signs and their severity, laboratory test results, and subsequent survival were collected by an experienced hospice team (comprising physicians and registered nurses) within 24 hours of hospice admission. When verbal communication with any patient was difficult due to the cognitive impairment, interviewers assessed these patients' status with their family caregiver. The proportion of unconscious patient in training group was 4.8%. (cognitive impairment = 3, 18/374 = 4.8%) and the proportion of unconscious patient in validation group was 8.2% (cognitive impairment = 3, 29/353 = 8.2%).

Almost all clinical symptoms and signs were collected according to those identified in previous studies [27-29]. Eighteen symptoms and signs were assessed, including pain, dyspnea, tiredness, heart rhythm (irregular versus regular), poor appetite (<500 cc of milk or <2 bowls of porridge by mouth or tube feeding within 24 hours of admission), medication for insomnia, nausea, vomiting, constipation, edema (scored as 0 = no; 1 = less than 1/2 finger breadth; 2 = 1/2 - 1 finger breadth; and 3 = >1 finger breadth), ascites (scored as 0 = no; 1 = only by ultrasound; 2 = shifting dullness by physical examination; 3 = umbilical protrusion), jaundice (scored as 0 = no; 1 = slightly yellowish; 2 = remarkably yellow; and 3 = deeply yellow or greenish), cognitive status (scored as 0 = clear; 1 = lethargy; 2 = confusion; 3 = comatose), performance status score according to the ECOG (range: 1-4), fever (core temperature ≥ 37.5°C), pressure sores, mean muscle power (sum of muscle power of each extremity divided by four, muscle powers graded using the Medical Research Council (MRC) scale of 0-5: 5 = normal power, 4 = moderate movement against resistance, 3 = movement against gravity but not against resistance, 2 = movement with gravity eliminated, 1 = flicker of movement, 0 = no movement), naso-gastric tube, and intervention tube placement (e.g., percutaneus nephrostomy (PCN), percutaneous transhepatic cholangio drainage (PTCD), pig tail for pleural effusion or ascites drainage, jejunostomy tube and percutaneous endoscopic gastrostomy tube). An additional 12 laboratory items were examined: complete blood count (e.g., white blood cell (WBC) count (normal range: male: 3.8-9.8*10^3^/μL, female: 3.6-9.6*10^3^/μL) and differential percentages, hemoglobin (normal range: male: 13-18 g/dL, female: 12-16 g/dL), and platelet (normal range: 120-320*10^3^/μL); and biochemistry examination: blood urea nitrogen (BUN) (normal range: ≤20 mg/dL), creatinine (normal range: ≤1.2 mg/dL), serum glutamic oxaloacetic transaminase (SGOT) (normal range: ≤38 IU/L), serum glutamic pyruvate transaminase (SGPT) (normal range: ≤41 IU/L), total bilirubin (normal range: ≤1.0 mg/dL), albumin (normal range: 3.4-4.8 g/dL), corrected calcium (normal range: 2.1-2.55 mmol/L), and blood sugar (normal range:70-110 mg/dL). Duration of survival days, which was defined as the period (in days) from the date of a hospice ward admitted to the date of death, or the end of follow-up, were also recorded.

The research team extracted independent prognostic factors from the 375 patients in the training set to establish a predictor model and then employed that predictor model to test the 353 patients in the validation group. The endpoint consisted of factors associated with the patient's expectation of dying within 7 days.

### Data Analysis

Comparability between the 2 groups was tested using Mann-Whitney-U test for skewed variables and Chi-square test for categorical variables. Nonparametric variables were represented as median (inter-quartile range) and categorical data were represented by number (n) and percentage (%). The survival rates were calculated by the Kaplan-Meier method and the differences between the survival curves were examined by the log-rank test. Univariate logistic regression analysis was performed to analyze the odds ratio of significant factors associated with patients who expired within 7 days. Variables having a *p *value < 0.05 in the univariate analysis were selected and evaluated by multivariate logistic regression models. Furthermore, a receiver operating characteristic (ROC) curve was employed in the training group to obtain the area under the curve (AUC), sensitivity, specificity, PPV and negative predictive value (NPV). All statistic assessments are two sided and evaluated at the 0.05 level of significance. Statistic analyses were performed using SPSS 15.0 statistics software (SPSS Inc, Chicago, IL).

## Results

**Figure 1:**
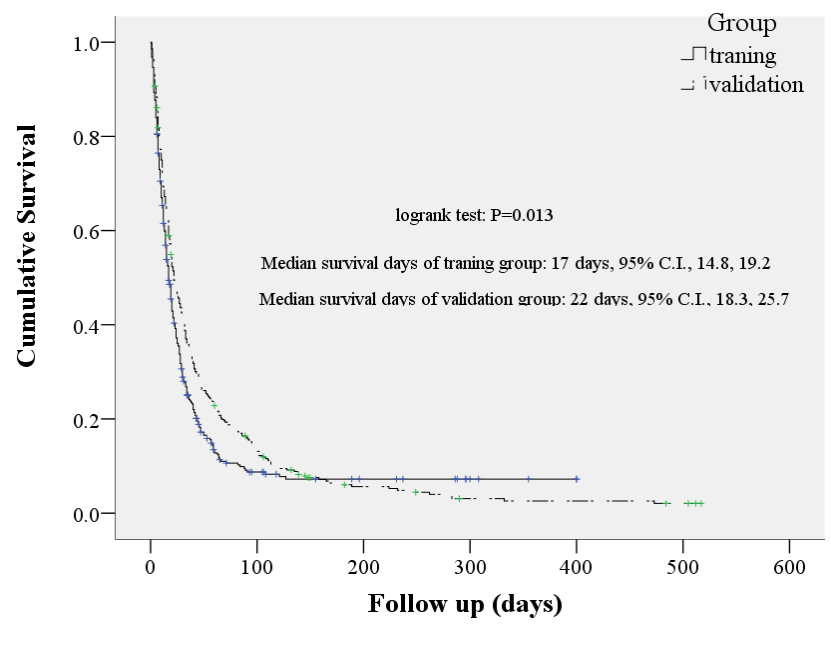
**Survival curve of the training and validation groups**.

The demographic characteristics of the training and validation groups are shown in Table 1. The training group had a median age of 67 (inter-quartile range = 58, 75); lung cancer was the most common diagnosis cancer (20.6%), 51.1% received chemotherapy as the most frequent type of treatment and the group was 61.0% male. The validation group had the same median age of 67 (inter-quartile range = 58, 75); liver cancer was the most common cancer diagnosis (20.1%), 53.8% received chemotherapy as the most frequent type of treatment and the group was 58.1% male. The groups were statistically significantly different in frequency of diagnosis of diabetes mellitus, incidence of head neck cancer, having radiotherapy treatment, and level of creatinine and albumin (*P *< 0.05). The survival rates were significantly different between the training and validation groups. The median survival dates were 17 and 22 days for the training and validation groups, respectively (Figure 1). Table 2 shows the prevalence of significant clinical signs by severity. The training and validation groups differed significantly in cognitive status, ECOG score, and ascites (*P *< 0.05).

**Table 1:** Patient characteristics in the training and validate sets.

	**Training (n = 374)**	**Validation (n = 353)**	***P*-value**
Age (years)	67 (54, 75)	67 (58, 75)	0.318
Gender			0.428
Male	228 (61.0%)	205 (58.1%)	
Female	146 (39.0%)	148 (41.9%)	
Diabetes mellitus	92 (24.6%)	120 (34.2%)	0.005*
Hypertension	151 (40.4%)	148 (41.9%)	0.671
Diagnosis			
Lung cancer	77 (20.6%)	58 (16.4%)	0.150
Liver cancer	74 (19.8%)	71 (20.1%)	0.912
Colon cancer	44 (11.8%)	42 (11.9%)	0.956
Stomach cancer	18 (4.8%)	25 (7.1%)	0.195
Head Neck cancer	41 (11.0%)	58 (16.4%)	0.032*
Pancreas cancer	16 (4.3%)	14 (4.0%)	0.833
Male GU cancer	16 (4.3%)	11 (3.1%)	0.408
Female GU cancer	27 (7.2%)	22 (6.2%)	0.596
Breast cancer	16 (4.3%)	9 (2.5%)	0.201
Esophagus cancer	11 (2.9%)	12 (3.4%)	0.833
Unknown and others	46 (12.3%)	50 (14.2%)	0.458
Treatment			
Operation	154 (41.2%)	165 (46.9%)	0.122
Chemotherapy	191 (51.1%)	190 (53.8%)	0.457
Radiotherapy	116 (31.0%)	143 (40.5%)	0.008*
Laboratory Parameters			
WBC (*1000/cumm)	10.01 (7.05, 15.19)	10.41 (7.34, 14.93)^†^	0.824
Hemoglobin (g/dl)	10.3 (9.1, 11.7)	10.6 (8.9, 11.9)^†^	0.340
Platelet (*1000/cumm)	210 (128, 312)	222 (138, 331)^†^	0.309
Glucose (mg/dl)	117 (99, 147)	118 (97, 156)^†^	0.941
BUN (mg/dl)	21 (15, 32)	19 (12, 32)^†^	0.067
Creatinine (mg/dl)	0.9 (0.7, 1.4)	0.7 (0.5, 1.1)^†^	<0.001*
SGOT (IU/L)	34 (20, 70)	35 (22, 81)^†^	0.335
SGPT (IU/L)	23 (14, 46)	26 (15, 47)^†^	0.382
Albumin (g/dl)	3.1 (2.7, 3.4)	2.8 (2.4, 3.2)^†^	<0.001*

* Statistically significant.

BUN = blood urea nitrogen; SGOT = serum glutamic oxaloacetic transaminase; SGPT = serum glutamic pyruvate transaminase.

^† ^Missing values were observed in this variable in the validation set.

**Table 2:** Prevalence of significant clinical signs by severity.

**Clinical signs**	**Training(n = 374)**	**Validation(n = 353)**	***P*-value**
Cognitive			0.014*
0	273 (73.0%)	228 (64.6%)	
1 to 3	101 (27.0%)	125 (35.4%)	
Edema			0.508
0	190 (50.8%)	188 (53.3%)	
1 to 3	184 (49.2%)	165 (46.7%)	
Jaundice			0.747
0	264 (70.6%)	253 (71.7%)	
1 to 3	110 (29.4%)	100 (28.3%)	
ECOG score			<0.001*
1 and 2	167 (44.7%)	26 (7.4%)	
3 and 4	207 (55.3%)	327 (92.6%)	
Ascites			<0.001*
0	218 (58.3%)	265 (75.1%)	
1 to 3	156 (41.7%)	88 (24.9%)	

* Statistically significant.

Table 3 presents the results of univariate and multivariate analysis of determinants of whether a patient would die within the next 7 days. The univariate logistic regression model indicated the following prognostic factors: diagnosis of liver cancer, cognitive status, edema, jaundice, ECOG score, ascites, WBC, platelet, BUN, creatinine, SGOP, SGPT, albumin level and respiratory rate (*P *< 0.05). Variables having a *P *value < 0.05 in the univariate analysis were selected and evaluated by multivariate logistic regression models. Multivariate logistic regression indicated that cognitive status (1 to 3 vs. 0, OR: 2.29, *P *= 0.014), edema (1 to 3 vs. 0, OR: 1.94, *P *= 0.038), ECOG score (3 and 4 vs. 1 and 2, OR: 3.45, *P *= 0.001), BUN and respiratory rate (on a linear scale, OR: 1.12, *P *= 0.004) were significantly associated with the likelihood of patients dying within 7 days.

**Table 3:** Determinants of patients who died within 7 days in the training set (n = 374).

	**Univariate analysis**			**Multivariate analysis**		
	**O.R**.	**95%CI**	***P*-value**	**O.R**.	**95%CI**	***P*-value**
Age (years)	1.01	(0.99, 1.03)	0.272			
Gender (male vs. female)	1.53	(0.92, 2.54)	0.099			
Diabetes mellitus (yes vs. no)	0.98	(0.52, 1.85)	0.953			
Hypertension (yes vs. no)	0.68	(0.36, 1.26)	0.215			
Cancer diagnosis						
Lung cancer (yes vs. no)	1.27	(0.72, 2.25)	0.411			
Liver cancer (yes vs. no)	2.22	(1.28, 3.86)	0.004*	1.63	(0.76, 3.50)	0.212
HN** cancer (yes vs. no)	0.63	(0.27, 1.48)	0.292			
Treatment						
Operation (yes vs. no)	0.66	(0.40, 1.09)	0.107			
Chemotherapy (yes vs. no)	1.27	(0.78, 2.05)	0.337			
Radiotherapy (yes vs. no)	0.87	(0.51, 1.47)	0.590			
Clinical signs						
Cognitive (1 to 3 vs. 0)	3.62	(2.18, 6.02)	<0.001*	2.29	(1.18, 4.43)	0.014*
Edema (1 to 3 vs. 0)	2.32	(1.42, 3.82)	0.001*	1.94	(1.04, 3.62)	0.038*
Jaundice (1 to 3 vs. 0)	2.23	(1.35, 3.67)	0.002*	1.00	(0.47, 2.15)	0.999
ECOG score (3, 4 vs. 1, 2)	6.13	(3.31, 11.37)	<0.001*	3.45	(1.65, 7.19)	0.001*
Ascites (1 to 3 vs. 0)	1.94	(1.20, 3.15)	0.007*	1.01	(0.49, 2.11)	0.975
Laboratory Parameters						
WBC (*1000/cumm)	1.03	(1.01, 1.05)	0.012*	1.02	(1.00, 1.04)	0.095
Hemoglobin (g/dl)	1.05	(0.93, 1.17)	0.445			
Platelet (*1000/cumm)	1.00	(1.00, 1.01)	0.031*	1.00	(1.00, 1.01)	0.145
Glucose (mg/dl)	1.00	(0.99, 1.00)	0.350			
BUN (mg/dl)	1.03	(1.02, 1.04)	<0.001*	1.02	(1.00, 1.03)	0.017
Creatinine (mg/dl)	1.46	(1.18 1.80)	<0.001*	1.00	(0.71, 1.41)	0.993
SGOT (IU/L)	1.00	(1.00, 1.01)	0.002*	1.00	(1.00, 1.01)	0.155
SGPT (IU/L)	1.01	(1.00, 1.01)	0.016*	1.00	(0.99, 1.01)	0.806
Albumin (g/dl)	0.56	(0.35 0.90)	0.016*	0.88	(0.50, 1.56)	0.666
Respiratory rate	1.12	(1.01, 1.19)	<0.001*	1.12	(1.04, 1.20)	0.004*

* Statistically significant.

** HN: Head Neck.

From this information we used variables identified by multivariate logistic regression to construct the following formula for the predictor model:

log [probability of dying within 7 days/(1- probability of dying within 7 days)] = [- 5.37 + 0.864* cognitive status (1 if cognitive = 0, 0 if otherwise) + 0.782* edema (1 if edema = 0, 0 if otherwise) + 1.208* ECOG (1 if ECOG = 1 and 2, 0 if otherwise) + 0.022*BUN + 0.104*respiratory rate].

**Figure 2:**
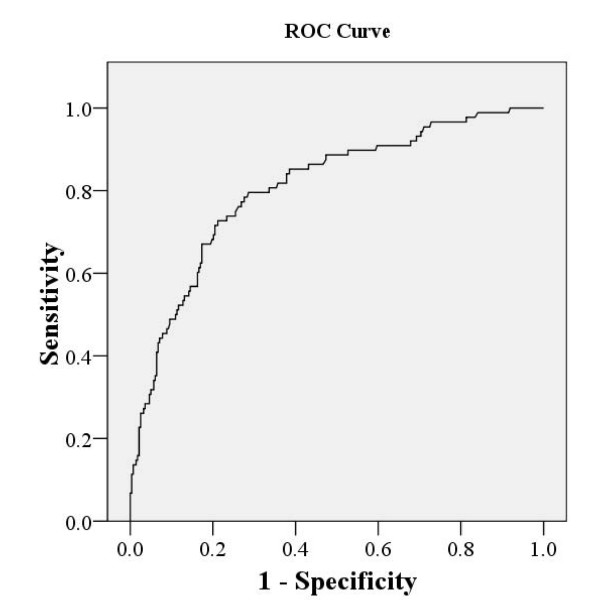
**ROC curve of training group**.

Figure 2 shows the ROC curve of the training group from which the cut-off point was chosen. The area under the curve (AUC) was 0.81 (*P *< 0.001, 95% CI: 0.76 to 0.86). The researchers decided to designate the cut-off point as probability of 0.2 in both training and validation groups. Table 4 presents the sensitivity and specificity of the model. When probability was >0.2, 7 days' survival was predicted for terminal patients in the training group and the validation group, with sensitivity of 80.9% and 71.0%, respectively; specificity of 65.9% and 57.7%, respectively; PPV of 42.6% and 26.8%, respectively; NPV of 91.7% and 90.1% (Table 4).

**Table 4:** Sensitivity and specificity of cut-off prognostic survival scale for patients dying within seven days in both training and testing samples.

	**Training (n = 374)**	Validation* (n = 349)
Sensitivity	80.9%	71.0%
Specificity	65.9%	57.7%
PPV	42.6%	26.8%
NPV	91.7%	90.1%

PPV: Positive Predictive Value; NPV: Negative Predictive Value

* Missing data in the validation group: 1 in data for BUN and 3 in data for Respiration rate.

Using this cut-off point, we observed 80.9% sensitivity and 65.9% specificity to predict whether the terminal cancer patients died within 7 days. The PPV for predicting death within 7 days was 42.6%, but the NPV for not dying within 7 days was 91.7%, suggesting that the model may be more reliable for predicting the 7-day survival. The same model was subsequently validated in a separated group of terminal patients. We observed 71.0% sensitivity and 57.7% specificity to predict the patients who died within 7 days. The PPV for predicting death within 7 days was 26.8%, and the NPV for not dying within 7 days was 90.1%. All these parameters of validation group were lower than those of training group, but sensitivity was still over 70% and the model also showed more reliability for predicting 7-day survival than death within 7 days.

## Discussion

The present study proposes a predictor model to estimate probability of 7-day survival for patients in terminal-stage cancer. The five predictors identified by multivariate logistic regression analysis were patient's cognitive status, edema, ECOG performance status, BUN and respiratory rate. A formula of the predictor model including these five predictors was developed and tested in both the training and validation groups. The cut-off point of probability of 0.2 was chosen based on the ROC curve of the training group. In our study, the area under the curve (AUC) was 0.81 (*P *< 0.001, 95% CI: 0.76 to 0.86). When the probability was >0.2, terminal patients died within 7 days was predicted in the training group and the validation group, with sensitivity of 80.9% and 71.0%, specificity of 65.9% and 57.7%, PPV of 42.6% and 26.8%, and NPV of 91.7% and 90.1%, respectively.

The predictors in our model agreed with other most recent models. ECOG performance status was a strong predictor (odd ratio = 6.13 in univariate logistic model, and 3.45 in multivariate analysis), as in Chuang's and Bozcuk's reports [16,17]. Cognitive score, another strong predictor (odd ratio = 3.62 in univariate logistic model, and 2.29 in multivariate analysis), was also identified by Bruera and Chuang [10,16]. Like us, Morita and Chuang identified edema as a significant predictor for length of survival among terminal cancer patients [11,16]. Accuracy of our study, expressed by ROC area (AUC), was comparable to that of Bozcuk [17].

The uniqueness of our model was the inclusion of blood urea nitrogen (BUN), and respiratory rate, one of the basic vital signs, in the formula. The study shows that BUN is more significant for survival than creatinine. Elevation of BUN is termed azotemia. Terminal azotemia means the state of terminal dehydration, gastrointestinal bleeding, and others of patients [30]. Only Chung attempted to predict survival within the one week window. We included fewer predictors than Chuang [16], whose eight characteristics include liver and lung metastases. Borzuk showed a similar area under the curve to ours, but only tested likelihood of dying in the hospital, not death within seven days [17]. We also found that the higher the respiratory rate, the greater the possibility of survival of less than one week.

This scale is an important improvement over previous methods. First, every terminally ill cancer patient receives the probability of 7-day survival within 24 hours of admission by our prognostic scale. The reader can use the method indicated in the **supplementary information **to calculate the probability. Second, we have identified a laboratory factor (BUN) and patient vital sign (respiratory rate) as additional prognostic parameters. Thirdly, this prognostic scale shows relatively high sensitivity (80.9%) and NPV (91.7%) for predicting 7-day survival among terminal cancer patients. This means that our prognostic scale has more value for identifying those patients not late referral for hospice care than for positively identifying those patients within 7 days of death.

Correct survival estimations in terminally ill cancer patients helps prevent inappropriate therapies, avoid actions that could worsen the patient's quality of life, and allows for support for patients and families by timely referred to hospice care. Knowing the approach of one's death can make for better decision-making and quality of life. Using Kellehear's model of spiritual needs, Leung et al. found that awareness of terminal illness was associated with well-being, even at the end of life [31]. Accurate prediction of survival is vital in planning effective palliative care and appropriately adjusting some medications [11]. Timely referral was associated with greater satisfaction in both patients and their family members [32].

Our study had some limitations. First, the degree of hospice and palliative care varies around the world, so including a representative population at similar and specific points during the course of their terminal illness is difficult [33]. Certain variables including survival and some clinical signs by severity (e.g., cognitive, ECOG score, ascites) were statistically different in both groups. However, this limitation shows that the participants of both groups were selected sequentially without bias, as in other studies [16,17]. Second, some data may inevitably introduce bias. For example, the data on weight lost were based on information provided by patients' relatives when the patient could not accurately recall body weight three months prior. In addition, a limited number of patients (8.2%) were assessed by family caregivers rather than by patients due to the cognitive impairment, which may have introduced some bias. Finally, a hospital-based study may not fully apply to community-based patients. In the present study, the number of patients meeting the inclusion criteria was limited in a community hospital setting. This could be an issue for further study.

In addition, the study has certain strengths. The NPV of our prognostic scale is 91.7%, and 90.1%, respectively, in the training and validation groups. This means that our prognostic scale would have more value for identifying those patients *not *late referral to hospice care than for positively identifying those patients with 7 days to death. Also, it provides a calculation (by R syntax or excel) for determining probability of survival within 7 days.

## Conclusion

This prognostic scale, including patient's cognitive status, edema, performance status, BUN and respiratory rate, showed a relatively high sensitivity and NPV for predicting 7-day survival among terminal cancer patients. We believe the proposed scale could be useful for predicting survival 7-day for terminal cancer patients, in addition, *not *late referral to hospice care.

## Competing interests

The authors declare that they have no competing interests.

## Authors' contributions

We declare that all the listed authors have participated actively in the study and all meet the requirements of the authorship. YHK carried out the epidemiology, and drafted the manuscript. NSL participated in its design and coordination. MHW participated in the data collection. JKC participated in the design of the study and performed the statistical analysis. SCC conceived of the study, and helped to draft the manuscript. All authors read and approved the final manuscript.

## Pre-publication history

The pre-publication history for this paper can be accessed here:


